# Spironolactone to prevent cardiovascular events in early-stage chronic kidney disease (STOP-CKD): study protocol for a randomized controlled pilot trial

**DOI:** 10.1186/1745-6215-15-158

**Published:** 2014-05-06

**Authors:** Khai P Ng, Poorva Jain, Gurdip Heer, Val Redman, Odette L Chagoury, George Dowswell, Sheila Greenfield, Nick Freemantle, Jonathan N Townend, Paramjit S Gill, Richard J McManus, Charles J Ferro

**Affiliations:** 1 Department of Renal Medicine, Queen Elizabeth Hospital Birmingham, Mindelsohn Way, Birmingham B15 2WB, UK; 2 Primary Care Clinical Sciences, School of Health and Population Sciences, University of Birmingham, Birmingham B15 2TT, UK; 3 Department of Primary Care and Population Health, UCL Medical School, Rowland Hill Street, London NW3 2PF, UK; 4 Department of Cardiology, Queen Elizabeth Hospital Birmingham, Birmingham B15 2WB, UK; 5 Department of Primary Care Health Sciences, University of Oxford, 2nd Floor, 23-38 Hythe Bridge Street, Oxford OX1 2ET, UK

**Keywords:** arterial stiffness, cardiovascular events, chronic kidney disease, feasibility, mineralocorticoid receptor antagonist, pulse wave velocity, qualitative, randomized controlled trial, renal dysfunction, spironolactone

## Abstract

**Background:**

Chronic kidney disease is associated with increased arterial stiffness even in the early stages and this is thought to be a key mediator in the pathophysiology of the increased cardiovascular risk associated with this condition. The use of low-dose spironolactone has previously been shown to improve arterial stiffness and reduce left ventricular mass safely in early-stage chronic kidney disease in the context of careful monitoring at a university hospital. However, the majority of patients with chronic kidney disease are managed by their general practitioners in the community. It is not known whether similar beneficial effects can be achieved safely using spironolactone in the primary care setting. The aim of this study is to determine whether low-dose spironolactone can safely lower arterial stiffness in patients with stage 3 chronic kidney disease in the primary care setting.

**Methods/design:**

STOP-CKD is a multicentre, prospective, randomized, double-blind, placebo-controlled pilot trial of 240 adult patients with stage 3 chronic kidney disease recruited from up to 20 general practices in South Birmingham, England. Participants will be randomly allocated using a secured web-based computer randomization system to receive either spironolactone 25 mg once daily or a matching inactive placebo for 40 weeks, followed by a wash-out period of 6 weeks. Investigators, outcome assessors, data analysts and participants will all be blinded to the treatment allocation. The primary endpoint is improved arterial stiffness, as measured by carotid-femoral pulse wave velocity between baseline and 40 weeks. The secondary endpoints are incidence of hyperkalaemia, change in estimated glomerular filtration rate, change in urine albumin:creatinine ratio, change in brachial blood pressure, change in pulse waveform characteristics and overall tolerability of spironolactone. An additional quality control study, aiming to compare the laboratory serum potassium results of samples processed via two methods (utilizing routine transport or centrifugation on site before rapid transport to the laboratory) for 100 participants and a qualitative research study exploring patients’ and general practitioners’ attitudes to research and the use of spironolactone in chronic kidney disease in the community setting will be embedded in this pilot study.

**Trial registration:**

Current Controlled Trials ISRCTN80658312.

## Background

Chronic kidney disease (CKD) is a long-term condition, which has been described as the gradual and usually permanent loss of kidney function over time [1]. The glomerular filtration rate (GFR) has become the most accepted test to assess kidney function and can be estimated using several equations. One of the most widely used equations to calculate and report estimated GFR (eGFR) was developed by the Modification of Diet in Renal Disease (MDRD) study group using four variables: age, ethnicity, sex and standardized serum creatinine levels calibrated to an assay traceable to isotope-dilution mass spectrometry [2]. This equation provides a measured eGFR of less than 90 ml/min per 1.73 m^2^ and allows reasonably accurate GFR estimation in patients with CKD. Since the introduction of a clearer, multilayered definition of the CKD based upon eGFR proposed by the National Kidney Foundation Kidney Disease Outcome Quality Initiative in 2002, there has been an increasing global awareness and recognition of this important health condition [3]. The prevalence of early CKD is increasing and now affects >13% of the population of the developed world [4,5]. For an important minority, CKD can progress to established renal failure (ERF), requiring renal replacement therapy in the form of either dialysis or transplantation [1]. The shift in focus for health services to active early recognition and management of chronic conditions has been a growing focus for health services in recent years.

There is now convincing evidence confirming CKD as an independent risk factor for cardiovascular (CV) morbidity and mortality [4,6-8]. Although the cardiovascular risk associated with ERF is substantial, the global health burden of cardiovascular morbidity and mortality caused by earlier stages of CKD may be much greater, given the much higher prevalence of > 13% in developed countries [3,9]. Patients with stage 3 CKD (an eGFR of 30 to 59 ml/min per 1.73 m^2^) represent the largest group, accounting for about 8% of the adult population [9]. These patients are mostly managed in the primary care setting [10,11] and are far more likely to die from adverse cardiovascular events than to progress to ERF [12]. Over a one-year period (2009 to 2010), there were approximately 7,000 excess myocardial infarctions and 12,000 excess strokes in patients with CKD in England compared with a non-CKD age- and sex-matched population [13]. The cost of these excess cardiovascular events to the UK National Health Service is estimated at £174 to £178 million [13].

In addition to the conventional CV risk factors (that is, hypertension, hypercholesterolaemia and smoking), other risk factors including activation of the renin-angiotensin-aldosterone system (RAAS), increased oxidative stress or inflammation, proteinuria and mineral bone disorder are also believed to be vital in contributing to the high CV burden in the CKD population [14,15]. Cardiovascular disease in CKD differs from that of the general population [14,15]. While vasculo-occlusive events, such as myocardial infarction, are prevalent in CKD and remain one of the important causes of death among the CKD population, a greater proportion of cardiovascular deaths in CKD are in fact attributable to sudden cardiac death, arrhythmia and congestive heart failure [16]. There are two distinct but overlapping arterial pathologies associated with CKD: atherosclerosis and arteriosclerosis [17]. Atherosclerosis is primarily an intimal disease characterized by patchy distribution of fibro-atheromatous plaques, leading to vascular occlusion. In contrast, arteriosclerosis is a diffuse disease of the arterial medial layer associated with increased collagen content, vascular calcification, hypertrophy and hyperplasia of vascular smooth muscle cells, resulting in thickening and hardening of the arteries [15]. Elastic arteries are important to buffer the pressure oscillations resulting from the intermittent ventricular ejection, providing a ‘cushioning function’ to supply steady blood flow to peripheral organs and tissues [18]. This loss of arterial distensibility in CKD is believed to be a key mechanistic pathway that leads to myocardial hypertrophy and fibrosis, systolic and diastolic cardiac dysfunction, increased risk of stroke and progressive kidney disease due to the exposure of increased systolic pressure and excessive perfusion pressure fluctuations to the myocardial, cerebral and renal microvasculature [15]. Notably, many of these abnormalities are evident even in patients with early CKD (stage 2 and 3) [19,20] despite satisfactory blood pressure (BP) control [21].

Despite the heightened CV risk in patients with CKD, there is a paucity of information to guide management [22], with guidelines often based on *post-hoc* or subgroup analyses of studies in the general population, which might be prone to bias [23]. Applying treatment strategies verified in the general population to patients with CKD is a highly debatable approach for several reasons, including the unique CV pathophysiology and risk profile [24]. Because of their well-documented advantages, randomized controlled trials (RCTs) represent the gold standard for testing hypotheses in medical research [25,26]. Studies in patients with CKD in the past have often produced negative or neutral results, possibly because of several pivotal methodological flaws [27]. These studies have often been underpowered as a consequence of ‘over-optimistic’ assumptions about event rates and the impact of therapeutic interventions. These factors need to be taken into account when planning future trials. Information gleaned from good quality, rigorously conducted pilot studies is essential when designing large, adequately powered hard-endpoint studies [28].

Thus far, the RAAS remains a major target for CV intervention, and inhibitors of this system have been used effectively in improving hypertension and proteinuria in patients with CKD [29-31]. In addition, retrospective analyses of the HOPE and PROGRESS data [19,32] suggested that angiotensin-converting enzyme inhibitors (ACEIs) might be even more effective in reducing CV risk in patients with evidence of CKD than in individuals with normal renal function. However prolonged use of ACEIs and angiotensin receptor blockers (ARBs) can lead to aldosterone ‘breakthrough’ and their efficacy in non-proteinuric CKD is less certain [29].

Aldosterone is a mineralocorticoid that is a key effector of the RAAS. *In vitro*, it is implicated in numerous CV effects, including endothelial dysfunction, transmural arterial inflammation, myocardial and vascular hypertrophy and fibrosis independent of BP control [33-36]. In human beings, a high aldosterone level is associated with higher left ventricular mass, increased arterial stiffness, endothelial dysfunction and insulin resistance [37]. The importance of increased arterial stiffness in CKD is evidenced by the strong independent association of this parameter with mortality in patients with CKD [38,39]. Treatment with mineralocorticoid receptor antagonists (MRAs) has been proven to confer a powerful prognostic benefit in patients with heart failure by the RALES and EPHESUS studies [40,41] and was confirmed in a meta-analysis [42]. In patients with CKD, MRAs are associated with significant decreases in proteinuria [43,44]. Additionally, in a randomized, double-blinded, placebo-controlled trial of 112 patients with stage 2 or 3 CKD in the secondary care setting, Edwards *et al.* demonstrated that the addition of spironolactone 25 mg once daily (a nonselective MRA) to background ACEI or ARB treatment safely and effectively reduced left ventricular mass (-14 ± 13 g versus +3 ± 11 g, *P* < 0.01) and decreased arterial stiffness, as measured by carotid-femoral pulse wave velocity (cfPWV: -0.8 ± 1.0 m/s versus -0.1 ± 0.9 m/s, *P* < 0.01), compared with placebo [45-47]. A trial to examine whether these desirable intermediate endpoints changes can be translated into long-term gains in terms of reduced CV morbidity and mortality in large CKD cohort is clearly warranted.

However, the majority of patients with stage 3 CKD in the UK [48] are managed in primary care by their general practitioners (GPs) and are often older with less well-defined renal phenotypes than the patients included in the hospital-based study. Furthermore, concerns among physicians about MRAs resulting in hyperkalaemia and worsened renal function, especially in the primary care setting, have also limited the widespread use of spironolactone in patients with CKD. Hence, before undertaking a large, expensive and appropriately powered definitive trial, this pilot study aims to carry out preliminary development work for and to test the feasibility of a RCT in the primary care setting. The primary objective is to determine the effect of spironolactone on arterial stiffness in nondiabetic patients with stage 3 CKD managed in primary care. The secondary objectives are to determine the safety of spironolactone in CKD stage 3; examine whether the rate of hyperkalaemia is affected by the method of laboratory analysis; assess the effect of low-dose spironolactone on BP and albuminuria in stage 3 CKD and qualitatively explore patients’ and healthcare professionals’ attitudes towards research in CKD and potential barriers to the use of spironolactone in CKD in a community setting. Patients with diabetes mellitus are excluded for a number of reasons. The pathophysiology of arterial stiffness might be different, with a greater importance of advanced glycation end-products, for example [15]. Furthermore, vascular calcification is more common and thus arterial stiffness may be less likely to improve with spironolactone [15]. Hyperkalaemia is more common in patients with CKD and diabetes than without; and this may be markedly worsened by spironolactone. Diabetes would be expected to affect 20 to 30% of a community sample of CKD, and hence would form a large subgroup within the trial. Thus, although diabetes is an important issue in CKD, this would be best explored in a separate study concentrating on diabetes rather than affecting the risk:benefit ratio of the proposed study in terms of reduced chance of outcome and a greater number of adverse events than expected.

## Methods

### Hypothesis

Low-dose spironolactone decreases arterial stiffness in patients with stage 3 CKD. The objectives of the study are detailed in Table 1.

**Table 1:** STOP-CKD study objectives

Pilot study		To determine the recruitment rate and feasibility of the study
Quantitative arm	Primary	To determine the effect of spironolactone on arterial stiffness in patients with stage 3 CKD
	Secondary	To determine the safety of spironolactone in stage 3 CKD in a primary care setting, in regards to the incidence of hyperkalaemia, worsened renal function and other adverse events
		To assess the effect of spironolactone on blood pressure and albuminuria in stage 3 CKD
		To assess the effect of spironolactone on pulse wave characteristics
Potassium substudy		To examine whether the different methods of serum processing affect the rate of hyperkalaemia seen in primary care
Qualitative arm		To examine patients’ and healthcare professionals’ attitudes towards CKD and research in CKD in the community setting
		Explore patients’ and healthcare professionals’ attitudes towards the use of spironolactone in CKD in a community setting and potential barriers to its use

## Design

This is a multicentre, prospective, randomized, placebo-controlled, double-blinded pilot trial in patients with stage 3 CKD. Patients registered in participating primary care practices within South Birmingham, England will be screened with a view to recruiting 240 eligible participants. Potential participants will be identified by searching computerized primary care clinical records for patients with biochemical evidence of stage 3 CKD (defined as an eGFR of 30 to 59 ml/min per 1.73 m^2^). The GFR will be estimated by the four-variable MDRD formula with serum creatinine recalibrated to be traceable to an isotope-derived mass spectroscopy method [49]. Potentially eligible patients will receive a patient information sheet (Additional file 1) and be invited to attend a screening visit at their own general practice, where the study will be explained further. Written consent will be obtained by the research team prior to enrolment into the study (Additional file 2). Following the screening visit, all recruited eligible, consenting participants will be randomized to receive either placebo or spironolactone 25 mg once daily orally for 40 weeks. Participants will be followed up during the duration of treatment and for 6 weeks after discontinuation of trial medication (wash-out period). The total study duration is 46 weeks. The cfPWV will be measured using a Vicorder (Skidmore, Bristol, UK) system [50] at baseline, week 40 and week 46 to detect any change in arterial stiffness. Outcomes will be analyzed using an intention-to-treat analysis.

Ethical approval has been received from the National Research Ethics Service West Midlands Coventry and Warwickshire (Reference No 12/WM/0168) and clinical trial authorization has been granted by the Medicine and Healthcare Products Regulatory Agency (MHRA) (Reference No 21761/0274/001-0001). The research team will seek further approval from the National Research Ethics Service and the MHRA for any protocol modifications. The sponsor, investigators, trial steering committee (TSC), data management committee (DMC), coordinating centre, recruiting sites, all members of the study team and all trial participants will be informed of the modifications.

The study will be coordinated by the Primary Care Clinical Research and Trials Unit (PC-CRTU), which has been fully accredited by the National Institute for Health Research (NIHR) as a trials unit at the University of Birmingham according to the current guidelines for Good Clinical Practice. The study will be monitored to confirm compliance with the protocol and the protection of patients’ rights, as detailed in the Declaration of Helsinki. Informed written consent will be obtained from all participants. The inclusion criteria are that patients must be over 18 years old and have stage 3 CKD. Patients will be excluded for any of the following criteria:

• Diabetes mellitus,

• Terminal disease or considered otherwise unsuitable by GP,

• Clinical diagnosis of chronic heart failure,

• Atrial fibrillation,

• Alcohol or drug abuse,

• Inability to comply with trial medication and follow-up,

• Documented previous hyperkalaemia or intolerance of spironolactone,

• Documented Addisonian crisis or taking fludrocortisone,

• Severe hypertension: BP ≥ 180/110 mmHg,

• Systolic BP < 120 mmHg,

• Recent acute kidney injury or hospital admission (within previous 6 weeks),

• Chronic diarrhoea,

• Urine albumin:creatinine ratio (uACR) ≥ 70 mg/mmol,

• Serum potassium ≥ 5 mEq/l on screening visit,

• Concomitant co-trimoxazole medication,

• Concomitant angiotensin-converting enzyme inhibitor and angiotensin II receptor blocker medication,

• Concomitant lithium medication,

• Concomitant warfarin medication,

• Pregnancy,

• Breastfeeding,

• Planned major surgical intervention within 46 weeks of recruitment.

### Study procedure

**Figure 1:**
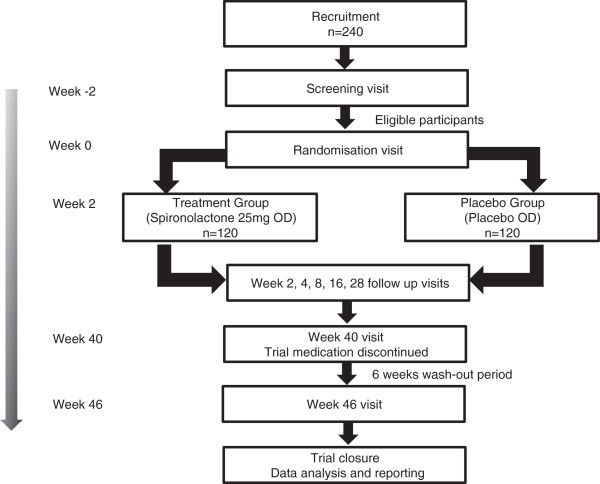
Study timeline.

The study timeline and schedule of follow-ups and assessments are summarized in Figure 1 and Table 2.

**Table 2:** Flowchart of assessment

			**Treatment**						
**Visit (week)**	**Screening**	**Randomization**	**2**	**4**	**8**	**16**	**28**	**40**	**46**
Valid informed consent gained	√	√							
Full demographic details	√								
Relevant medical history taken	√	√							
Concomitant medications	√	√	√	√	√	√	√		
Anthropometric measurements		√							
Blood pressure measurement	√	√	√	√	√	√	√	√	√
Pulse wave velocity and pulse waveform analysis measurement		√						√	√
Haematological and full biochemical profile	√		√					√	√
Renal profile				√	√	√	√		
Urine albumin:creatinine ratio	√							√	√
EQ5D-5 L questionnaire		√						√	
Medication monitoring questionnaire		√	√	√	√	√	√	√	√

EQ5D-5 L, European Quality of Life, 5 Dimensions, 5 Levels.

#### *Screening visit*

All consenting participants will attend a screening visit during which the following will be carried out: (i) completion of a questionnaire regarding demographic details, relevant medical history and concomitant medication; (ii) BP measurement noninvasively using an automated BpTRU machine (BPM-100, BpTRU™) [51]; (iii) blood and urine sampling. Estimated GFR on this screening visit will confirm the diagnosis of stage 3 CKD (two eGFR measurements of 30 to 59 ml/min per 1.73 m^2^ at least 90 days apart). A urine test will be used to exclude patients who have a urine albumin:creatinine ratio (uACR) > 70 mg/mmol. Participants with BP >140/90 mmHg and a uACR of 30 to 69 mg/mmol but not receiving either ACEI or ARB will be referred to their GP to be considered for ACEI or ARB treatment. They will be re-invited to the screening visit after at least 6 weeks treatment with ACEIs or ARBs.

#### *Randomization visit*

Eligible patients will then be invited back no later than two weeks after their initial visit to attend a randomization clinic. Informed consent will be sought again before randomization to commence trial medication (Additional file 3). Consenting participants will undergo the following assessments: (i) EQ5D-5 L (European Quality of Life, 5 Dimensions, 5 Levels) [52] (quality of life survey) and medication monitoring questionnaires (Additional file 4); (ii) anthropometric measurements; (iii) BP measurement; (iv) cfPWV measurement and pulse wave analysis (PWA). All participants will then be randomized to receive either inactive placebo or spironolactone 25 mg once daily orally for 40 weeks.

#### *Follow-up visits*

**Figure 2:**
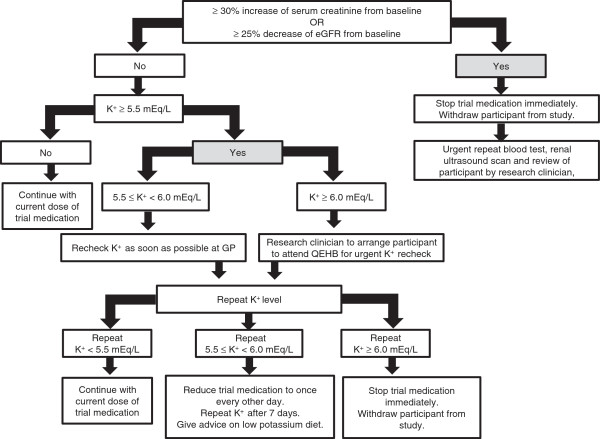
**Study flowchart on management of renal dysfunction and hyperkalaemia.** eGFR, estimated glomerular filtration rate; K^+^, serum potassium concentration.

Following randomization, all participants will attend follow-up visits at weeks 2, 4, 8, 16 and 28. The medication monitoring questionnaire will be filled in and brachial BP measurements as well as blood samples to monitor serum electrolytes and renal function will be taken at each visit. Participants with a persistently elevated BP of more than 150/90 mmHg will be referred to their GP for BP management according to the guidelines of the National Institute for Health and Care Excellence [53]. Participants with hyperkalaemia or renal function deterioration during the follow-up visits will be managed according to the study protocol (Figure 2).

All measurements performed at the screening and randomization visits will be repeated at 40 weeks after the randomization, marking the end of the treatment phase. All participants will discontinue the trial medication and adherence will be assessed via pill count. After a wash-out period of 6 weeks, all participants will attend final follow-up visits (week 46 visit), whereby all measurements performed at the week 40 visits will be repeated.

### Study assessments

#### *Blood pressure measurement*

Blood pressure will be measured using the BpTRU™ BPM-100 automated blood pressure monitor [51]. During the screening visit, six serial sitting blood pressure measurements will be taken simultaneously on both arms, to identify which arm to use for BP monitoring for all future visits. These six BP measurements will be performed via the automated machine at 1 minute intervals. Each BP reading will be recorded. The average BP reading on each arm will be derived from the mean of the second, third, fourth, fifth and sixth readings. If there is >20 mmHg difference in systolic BP or >10 mmHg difference in diastolic BP on the average BP reading between the arms, the arm with the higher reading will be selected for all future BP and Vicorder measurements. If not, the nondominant arm will be the selected measured arm. After measurement of sitting BP, postural BP will be measured after asking the participant to stand up for 1 minute from sitting position. Postural hypotension is defined as a drop of systolic BP >20 mmHg on standing. Serial sitting BpTRU BP measurements and postural BP will be repeated during each follow-up visit on weeks 2, 4, 8, 16, 28, 40 and 46.

#### *Carotid-femoral pulse wave velocity and pulse wave analysis measurement*

The Vicorder system provides a noninvasive non-operator-dependent method of obtaining cfPWV and PWA using a volume displacement technique. Participants will be lying supine at approximately 30° with the head and shoulders supported by a pillow to prevent flexion of the neck. The cfPWV and PWA measurements will be performed on the same side as for BP for each participant after 5 minutes of rest.

Measurements will be obtained by placing a 100-mm-wide BP cuff on the proximal thigh to measure the femoral pulse and a 30-mm-wide partial cuff on the neck at the level of the carotid artery. The distance between the midclavicular point and the middle of the thigh cuff will be measured and input to the Vicorder device as aortic path length. The cuffs will each inflate to 60 mmHg and the corresponding oscillometric signal of at least 14 s duration from each cuff will be digitally analyzed to extract, in real time, the pulse waveforms, pulse transit time and consequent cfPWV, which is a measure of arterial stiffness. Pulse wave analysis will be performed in a similar manner by placing the 100-mm-wide BP cuffs on the selected arm and proximal thigh. The cfPWV and PWA measurements will be performed in triplicate. The mean value of the three recordings will be used for subsequent analysis. Inconsistent values among the three recordings will be further examined by a designated senior investigator not involved in taking the measurements, to determine the validity of each measurement.

### Randomization

Investigators, outcome assessors, data analysts and participants will all be blinded to the treatment allocation via the use of an apparently identical inert placebo and a central automated allocation procedure. Participants will be stratified by practice location, systolic BP (above or below 140 mmHg) and urine albuminuria (uACR above or below 30 mg/mmol) and assigned to either active treatment or inactive placebo using a minimization algorithm with a 70:30 weighted-coined approach if there is an imbalance. Randomization assignment will be centrally generated using the PC-CRTU secured web-based randomization system.

### Unblinding

Researchers will be unblinded after the completion of 46 weeks follow-up of all subjects and closure of the trial. The practice staff and all participants involved in the study, including those who had withdrawn, will also be informed of their trial medication allocation (spironolactone or inactive placebo). During the trial, code breaking will be avoided whenever possible. If emergency unblinding is deemed to be appropriate, research team members have access to break the randomization code. When assessing severe adverse events, it will be assumed that the participants receive spironolactone (not inactive placebo). If the event is thought to be related and that the knowledge of treatment will alter the medical management, unblinding will be performed via the PC-CRTU secured web-based code-breaking system with a paper-based code-breaking system back up.

### Treatment

STOP-CKD is a double-blinded, placebo-controlled, randomized trial involving two treatment arms: spironolactone 25 mg orally once daily and inactive placebo orally once daily. All participants will receive their allocated trial medication for 40 weeks. The trial medication is supplied by an authorized trial medication manufacturing unit and funded via an NIHR Research for Patient Benefit programme grant for this study. The trial medication was labelled and packaged by the trial medication manufacturing unit prior to delivery to a designated community pharmacy hub. The trial medication will be dispensed at the pharmacy hub and delivered to the local community pharmacy closest to the recruited general practice for collection by the participants.

All participants will complete the medication monitoring questionnaire at each visit to record any side effect related to the trial medication and self-report trial medication compliance during the 40 weeks treatment phase. If participant’s serum potassium concentration is 5.5 to 5.9 mEq/l on repeat samplings, the trial medication will be reduced from once daily to once every other day (see Figure 2).

### Withdrawal criteria

Participants will be withdrawn from the trial if they choose not to continue, if their GP considers that continued participation in the trial is inappropriate or if they are no longer eligible according to the withdrawal criteria listed in Table 3. Participants who withdraw from the trial will be asked if they are willing to attend a final research visit within 7 days of stopping the trial medication for blood and urine sampling, BP and Vicorder measurement and completion of the EQ5D-5 L and medication monitoring questionnaire. Data will be collected for an intention-to-treat analysis.

**Table 3:** Withdrawal criteria

**System**	**Adverse effect**	**Action**
Blood pressure	Hypotension	Withdraw trial medication if systolic blood pressure <100 mmHg or postural drop of systolic blood pressure >20 mmHg.
Metabolic	Hyperkalaemia	Withdraw trial medication if serum potassium ≥ 6 mEq/l on repeat sampling.
	Hyponatraemia	Withdraw trial medication if serum sodium <130 mEq/l on two occasions.
Renal	Renal dysfunction	Withdraw trial medication if serum creatinine increment ≥30% or eGFR reduction ≥25% from baseline.
Endocrine	Men: gynaecomastia, impotence, diminished libido	Withdraw trial medication if participant is intolerant of the side effect or effects.
	Women: hirsutism, oligomenorrhoea, amenorrhoea, menorrhagia, breast tenderness	
Nervous system	Headache	Withdraw trial medication if symptom persists for >1 week.
	Confusion, ataxia, drowsiness	Check postural blood pressure and serum sodium level. If postural blood pressure and serum sodium levels are within normal levels, but symptoms persist for >1 week, withdraw trial medication.
	Lethargy	Withdraw trial medication if symptom persists for >1 week.
Dermatological	Rash	Withdraw trial medication.
	Lichen planus, lupus-like syndrome	Withdraw trial medication.
Hypersensitivity	Anaphylaxis, contact dermatitis, eosinophilia	Withdraw trial medication immediately.
Gastrointestinal	General abdominal discomfort	Withdraw trial medication if persistent discomfort for >1 week.
	Diarrhoea or vomiting	Withdraw trial medication if persistent diarrhoea or vomiting for >3 days.
	Gastric or duodenal ulcer or bleeding	Withdraw trial medication.
Haematological	Agranulocytosis	Withdraw trial medication.
Hepatic	Hepatotoxicity (alanine transferase > 123 U/l or bilirubin > 44 μmol/l)	Withdraw trial medication.
Oncological	Animal studies suggested association between spironolactone with benign adenoma of the thyroid and testes, malignant breast tumours, hepatocellular carcinoma and leukemia	Withdraw trial medication.

### Endpoints

The primary endpoint of the study will be a change in cfPWV between baseline and 40 weeks. Secondary endpoints are: (i) change in brachial BP; (ii) change in eGFR; (iii) change in uACR; (iv) change in pulse waveform characteristics; (v) incidence of hyperkalaemia; (vi) incidence of renal dysfunction (increment of creatinine ≥30% or reduction of eGFR ≥25% from baseline); (vii) incidence of other adverse events.

### Sample size calculation and planned statistical analysis

In our recent study of the effect of spironolactone, the Chronic Renal Impairment in Birmingham II (CRIB II) study, the standard deviation of the change in cfPWV was 1.0 m/s in the active treatment group and 0.9 m/s in the control group. Hence, 100 subjects in each arm will provide 90% power with an *α* value of 0.05 to demonstrate a difference in change of cfPWV of 0.5 m/s between the active treatment and control groups. We aim to recruit 240 patients to account for an approximate drop-out rate of 20%, which will result in at least 200 patients completing this randomized control trial, with 100 patients in each arm (inactive placebo versus spironolactone).

The difference in the primary outcome between experimental conditions will be assessed using generalized mixed models. Repeated measures within a subject will be characterized as *R* side residual over-dispersion parameters, accounting for baseline values. Frequency differences will be tested using Fisher’s exact test. A prespecified multivariable analysis will examine the influence of change in systolic BP and other relevant factors on changes in the primary endpoint. Outcomes will be analyzed using intention-to-treat analysis. A *P* value of less than 0.05 will be considered statistically significant.

Data entry, coding, security, storage, access and quality assurance will be managed according to PC-CRTU policy [54]. Study investigators will have access to the final trial dataset.

### Trial management

The STOP-CKD study will be coordinated by the PC-CRTU at the University of Birmingham according to the current guidelines for Good Clinical Practice. The chief investigator takes overall responsibility for the conduct of study. Any delegated or devolved responsibility will be documented in a delegation log. An investigators group will meet monthly to provide oversight of the developing trial, with more frequent operational meeting of the chief investigator, trial manager and trial team as required.

A TSC will be appointed and will provide overall supervision for the trial, in particular: trial progress, protocol compliance, patient safety and review of updated information. The TSC will include the trial management group, two lay representatives and an independent chair who has expertise relevant to the study. The TSC will meet every 3 to 6 months, depending on the phase of the study.

An independent DMC for the trial will be responsible for the regular monitoring of trial data. The committee will consist of an independent secondary care clinician, an independent academic general practitioner and an independent statistician. The DMC will assess the progress of the trial and give advice on whether the accumulated data from the trial, together with the results from other relevant trials, justifies the continuing recruitment of further patients. The committee will meet in person or by teleconference prior to the trial commencement and then 3 and 6 months after initiation of the trial. The DMC will make confidential recommendations to the TSC as the decision-making committee for the trial (Additional file 5).

### Monitoring and safety assessments

Monitoring will be performed according to PC-CRTU policy and be conducted centrally and at each local recruitment sites. Any major problems identified during monitoring will be reported to the TSC. All records will be maintained in accordance with local regulations and in a manner that ensures security and confidentiality. All adverse events and severe adverse events will be recorded and followed up for the duration of the study or until resolution. Assessment of adverse events will be performed by the study investigators. All serious adverse events will be graded and reported to the sponsor. Any suspected unexpected serious adverse reactions will be reported to the sponsor, ethics committee and MHRA.

### Substudies

#### Quality control of laboratory tests

The time lapses from blood sampling to analysis of sample in the biochemistry laboratory in the Queen Elizabeth Hospital Birmingham will be recorded. At week 2, two serum separator tube (SST) samples will be obtained from up to 100 participants. One SST from each of the 100 participants will be centrifuged on site (at the recruited general practice) before sending to the biochemistry laboratory at the Queen Elizabeth Hospital Birmingham for potassium analysis. The other parallel SSTs from the same participants will be sent via the routine specimens transport from the general practices to the laboratory. The serum potassium results from each pair of SSTs will be compared to examine the influence of time delay during routine general practice specimens’ transport on serum potassium results.

#### *Qualitative research study to address development and feasibility issues*

A qualitative research study will be embedded in this pilot RCT to explore patients’ and GPs’ attitudes to CKD, research in CKD and use of spironolactone in CKD in the community setting, and to examine potential barriers for research participation as well as barriers to the use of spironolactone in the community. The outcome of this qualitative study will help to indicate how trial procedures might be modified for a future definitive trial and improve our understanding of the factors that might limit the prescription of spironolactone in community settings.

The data sampling technique includes a one-to-one interview and a focus group. Interviews will be performed at the beginning of the study with the aim of involving both patients and GPs who have agreed or declined to participate in the RCT. Focus groups will be organized after participants have completed the RCT. General practitioners and patients will receive patient information sheets on the qualitative study. Informed written consent will be obtained prior to participation in the qualitative research.

A grounded theory approach [55] will be used to guide sampling, data collection and analysis. Purposive sampling will allow for maximum variety of patient and GP characteristics. Up to 30 patients and 30 GPs will be selected for interview. Up to four focus groups will be organized among participating patients and GPs. Patient sampling will ensure representation of views from participants of different age, ethnicity, socio-economic status and sex. General practitioner sampling will ensure representation of different ages of GP, practice locations and practice sizes. Interviewing will continue until data saturation has been achieved. The interviews will be confidential and face-to-face using a topic prompt, which will be refined over the course of the initial interviews [56]. They will be undertaken by a research fellow, supervised by an experienced qualitative researcher. Both the interviews and focus group meetings will be carried out in a place convenient to the interviewees.

All interviews and focus groups will be audio-taped and transcribed verbatim. Transcriptions will be read and checked for accuracy by the researcher and the text entered into a computerized database using the NVivo (QSR International) qualitative software package [57]. Constant comparative analysis [55] will be used to interpret the data. Data collection and analysis will be iterative, with new data being used to confirm or challenge emerging concepts.

## Discussion

The deleterious effects of excessive aldosterone activation are well-established and known to promote adverse metabolic effects, vasoconstriction, inflammation and fibrosis, which ultimately lead to significant CV and renal damage. Substantial improvement of CV function and structure with the use of spironolactone in the RCT of Edwards *et al. *[45-47] has further demonstrated the potential of aldosterone inhibition as a powerful CV treatment for the CKD population. If the use of low-dose spironolactone is proven to achieve similar safety and desirable effects on arterial stiffness in the CKD population managed in primary care in this pilot study, it will undoubtedly facilitate the development of a future definitive large RCT to determine the effect of spironolactone on decisive hard-endpoints (CV morbidity and mortality).

## Trial status

Upon submission, the STOP-CKD study is in the process of patient recruitment.

### Protocol amendments

Version 1.0 approved on 22 March 2012.

Version 2.0 approved on 18 June 2012.

Version 3.0 approved on 26 July 2012.

Version 4.0 approved on 20 June 2013.

### Dissemination policy

We would anticipate publishing the overall findings of this study in peer-reviewed journals and disseminate the results to all trial participants. All publications and presentations relating to the trial will be authorized by the TSC and authorship eligibility will be applied according to the guidelines of the International Committee of Medical Journal Editors. No use of professional writers is intended.

## Abbreviations

ACEI: angiotensin-converting enzyme inhibitor; ARB: angiotensin receptor blocker; BP: blood pressure; cfPWV: carotid-femoral pulse wave velocity; CKD: chronic kidney disease; CV: cardiovascular; DMC: data monitoringcommittee; eGFR: estimated glomerular filtration rate; EQ5D-5 L: European Quality of Life, 5 Dimensions, 5 Levels; ERF: established renal failure; GFR: glomerular filtration rate; GP: general practitioner; MDRD: Modification of Diet in Renal Disease; MHRA: Medicine and Healthcare Products Regulatory Agency; MRA: mineralocorticoid receptor antagonist; NIHR: National Institute for Health Research; PC-CRTU: Primary Care Clinical Research and Trial Unit, Birmingham; PWA: pulse wave analysis; RAAS: renin-angiotensin-aldosterone system; RCT: randomized controlled trial; SST: serum separator tube; TSC: trial steering committee; uACR: urine albumin:creatinine ratio.

## Competing interests

The authors declare that they have no competing interests.

## Authors’ contributions

KPN was involved in the study design, application for Research Ethical Committee, MHRA and NHS Research and Development approval, patient recruitment and drafting of the manuscript. PJ was involved in the study design and patient recruitment. GH was involved in patient recruitment. VR and OLC coordinated the study. GD and SG were involved in designing the qualitative aspect of the study. NF was the study statistician. JNT and PSG were involved in the study design. RJM was involved in the study design and reviewing the manuscript. CJF was the chief investigator and was involved in the study design and writing and reviewing the manuscript. All authors critically reviewed the manuscript and gave final approval of the version to be published.

## Authors’ information

KPN is a nephrology registrar at the Queen Elizabeth Hospital Birmingham and a PhD student at the University of Birmingham. PJ is a nephrology registrar at the Queen Elizabeth Hospital Birmingham and a PhD student at the University of Birmingham. GH is the study research nurse at the PC-CRTU in the University of Birmingham. VR is the trial coordinator at the PC-CRTU in the University of Birmingham. OLC is the senior trial manager of the PC-CRTU in the University of Birmingham. GD is a qualitative research fellow at the University of Birmingham. SG is Professor of Medical Sociology and a qualitative methodologist in Primary Care Clinical Sciences at the University of Birmingham. NF is Professor and Chair of Clinical Epidemiology and Biostatistics in the department of Primary Care & Population Health of University College London. JNT is Professor of Cardiology at the Queen Elizabeth Hospital Birmingham. PSG is a reader in primary care research and a general practitioner. RJM is Professor of Primary Care at the University of Oxford and a general practitioner. CJF is a consultant nephrologist at the Queen Elizabeth Hospital Birmingham.

## Supplementary Material

Additional file 1 Patient information sheet, version 2.2. Click here for file

Additional file 2 Consent form, part 1, version 2.2. Click here for file

Additional file 3 Consent form, part 2, version 2.2. Click here for file

Additional file 4 Medication monitoring questionnaire. Click here for file

Additional file 5 Data monitoring committee charter. Click here for file

## Declaration

This paper presents independent research funded by the NIHR under the Research for Patient Benefit programme (PB-PG-0110-21226). The views expressed in this publication are those of the authors and not necessarily those of the NHS, the NIHR or the Department of Health.
