# Supplementary material for: Spironolactone to prevent cardiovascular events in early-stage chronic kidney disease (STOP-CKD): study protocol for a randomized controlled pilot trial
Source: Trials. 2014 May 6;15:158. doi: 10.1186/1745-6215-15-158 (PMC4113230; doi:10.1186/1745-6215-15-158)
Supplement: Additional file 1 — Patient information sheet, version 2.2. [file 1745-6215-15-158-S1.pdf]

STOP-CKD

Spironolactone to Prevent Cardiovascular Events in
Early Stage Chronic Kidney Disease: A Pilot Trial Version 2.2 20/06/2013

Patient Information Sheet

We would like to invite you to take part in a research study. Before you decide, we would like you to understand why the research is being done and what it would involve for you. Please take time to read the following information carefully. Talk to others about the study if you wish. Ask us if there is anything that is not clear or if you would like more information. Take time to decide whether or not you wish to take part.

Part 1

<u>What is the purpose of the study?</u>

About 1 in 10 people have mild chronic kidney disease (CKD) most commonly because kidney function declines with age. Patients with kidney disease are at increased risk of hardening of the blood vessels, which can lead to heart disease and stroke. We are investigating a medication called Spironolactone which has been used to treat patients with high blood pressure, heart disease or liver disease for a long time. In patients with mild kidney disease in our specialist hospital kidney clinic, **Spironolactone** seems to improve heart function and reduces hardening of the blood vessels. We want to find out if Spironolactone has similar effects on patients with early kidney disease treated in the community.

<u>Do I have to take part?</u>

It is up to you to decide if you wish to join the study. If you agree to take part, we will then ask you to sign a consent form. You are free to withdraw at any time, without giving a reason. Your medical care will be unaffected whether or not you take part.

<u>Why have I been invited?</u>

You have been invited to take part in this research study because you have had a previous blood test at at your GP surgery that shows you may potentially have reduced kidney function.

<u>Who else is taking part?</u>

We are inviting people like you, from different GP surgeries in Birmingham to take part. We need to identify a total of 240 patients with early kidney disease to participate in this research study.

<u>What will happen to me if I take part?</u>

Participation in this study is **voluntary**. If you decide that you would like to take part in this study, you will be asked to attend a clinic at your own GP surgery run by the research team. A member of the team will explain the study to you and answer any questions you might have. They will ask you some questions about your general health and any medication you may be taking. You will then be asked to sign a consent form. You should only do this if you are happy that you understand the project and want to take part. A urine sample and 30ml blood sample (less than 3 tablespoons) will be taken to confirm if you have reduced kidney function by a kidney specialist and if you are eligible to take part in this study.

If you are eligible, you will then be contacted by the research team and invited back to another clinic, also at your surgery. A member of the team will discuss the study with you again and check that there have been no changes to your health or medication. We will check your blood pressure, measure your weight, height, waist, hip and thigh circumference and measure the stiffness of your blood vessels. You will be asked to complete a questionnaire. A computer will then decide at random (like tossing a coin) which type of treatment you will have. Half of the people taking part will be prescribed the medication and the other half will be prescribed a placebo capsule (A placebo capsule looks similar to the active medication but contains no active drug). You will be prescribed the medication the computer chooses for you using a prescription that can be collected from one of our designated pharmacies. This medication needs to be taken daily for 10 months.

Over the course of the study, you will be seen by the research team at regular intervals (2, 4, 8, 16, 28 weeks) at your own surgery. They will again take your blood pressure measurements, repeat blood samples tests and completion of a Quality of Life questionnaire. At 40 weeks, we will repeat the measurement of your blood vessels' stiffness and collect blood and urine samples. You will be informed to stop the trial medication after the 40 week visit. At the end of the study (6 weeks after the trial medication is stopped), we will asked you to attend a final clinic to have a repeat blood and urine test, as well as repeat measurement of blood vessels' stiffness. So in total, you will be seen at your surgery 8 times.

What will I have to do?

You will need to take the study medicine regularly during the course of the study (40 weeks) and attend all the study visits. We will ask you to report any missed tablets during the study period. You should also continue taking all of your normal tablets. We will review your medication before you start the study to make sure the study medication does not interfere with your usual tablets.

We will ask you to tell us if your GP starts you on any new medicines during the study. If your Potassium level is found to be high on the blood test during the study period, we might ask you to restrict certain foods in your diet to avoid foods high in potassium and provide you with an information sheet.

What happens at the end of the research study?

ONCE YOU HAVE COMPLETED ALL OF YOUR TESTS AT THE FINAL VISIT, THE TRIAL MEDICATION (SPIRONOLACTONE OR PLACEBO) WILL BE STOPPED. THE RESULTS WILL BE STUDIED AND ANALYSED. YOUR USUAL MEDICAL CARE WILL CONTINUE AS BEFORE ONCE THE STUDY IS COMPLETED. ALL SPECIMENS COLLECTED DURING THE STUDY WILL BE STORED FOR 5 YEARS AFTER THE COMPLETION OF THIS STUDY. IF YOU WOULD LIKE TO KNOW THE RESULTS WE WILL SEND YOU A SUMMARY.

Will I get paid for taking part?

We are unable to pay you for participating but we will reimburse your travel expenses to and from your GP surgery or Queen Elizabeth Hospital Birmingham.

What are the alternatives for treatment?

There are currently no other medicines licensed for reducing stiffness of the blood vessels in patients with early kidney disease.

What are the possible risks of taking part?

With the exception of the blood tests, study procedures should not cause any pain or discomfort. There are small risks of increased levels of salts in the blood, reduced kidney function or low blood pressure with the use of Spironolactone, requiring the withdrawal of the medication. However, the dose of the trial medication

is relatively low and you will be closely monitored by kidney specialists during the study to ensure that those risks are minimised.

Spironolactone should be avoided during pregnancy. If you are a woman of child-bearing age, we would ask you for permission to perform a pregnancy test prior to starting the trial medication and to agree not to become pregnant whilst taking it. If you do become pregnant during the course of the study, you must stop taking the trial medication immediately and inform our research team so an appropriate course of action can be taken.

WHAT ARE THE OTHER SIDE EFFECTS OF THE TREATMENT RECEIVED WHEN TAKING PART?

The most common side effects from Spironolactone are diarrhoea, drowsiness, headache, nausea, stomach cramping and vomiting. Such effects are usually mild and temporary and resolve when the drug is stopped.

Other less common but serious side effects are severe allergic reactions (rash, hives, itching, difficulty breathing, tightness in the chest, swelling of the mouth, face, lips, or tongue), black, tarry, or bloody stools, change in the amount of urine produced, confusion, dark urine, decreased sexual ability, enlarged breasts in men, irregular or missed menstrual periods, severe or persistent stomach pain, symptoms of abnormal fluid or electrolyte levels (i.e.: fast, slow, or irregular heartbeat, increased thirst, muscle weakness, severe or persistent dry mouth, nausea, or vomiting, severe or persistent dizziness or drowsiness, unusual fatigue or sluggishness, tingling sensation), yellowing of the skin or eyes.

If you were to experience these serious side effects, **you should stop taking the trial medication immediately and contact our research team on 0800 9230329**.

WHAT ARE THE BENEFITS OF TAKING PART?

Our previous research study showed that Spironolactone improves heart function and reduces hardening of the blood vessels in patients with mild kidney disease in our specialist hospital kidney clinic. However, we are unable to guarantee any direct benefit to you as a result of taking part in this study. Nonetheless, you will contribute to an improved understanding of Spironolactone and its effects on blood vessel and kidney disease. The information gained from this study will also contribute to further studies and may help improve the treatment of people with kidney disease in the future.

What if there is a problem?

Any complaint about the way you have been dealt with during the study or any possible harm you might suffer will be addressed. The detailed information on this is given in Part 2.

Will my taking part in the study be kept confidential?

Yes. We will follow ethical and legal practice and all information about you will be handled in confidence. The details are included in Part 2.

What will happen if I don't take part?

PARTICIPATION IS ENTIRELY VOLUNTARY. IF YOU DECIDE NOT TO TAKE PART THEN YOU WILL CONTINUE TO BE SEEN AS BEFORE IN OUTPATIENT CLINICS. A DECISION NOT TO TAKE PART WILL NOT AFFECT YOUR ROUTINE CARE IN ANY WAY.

If the information in Part 1 has interested you and you are considering participation, please read the additional information in Part 2 before making any decision.

Part 2

WHAT IF RELEVANT NEW INFORMATION BECOMES AVAILABLE?

Sometimes we get new information about the treatment being studied. If this new information means that we should stop the study, or change how we are running it, we will do this and make sure that you are offered the best treatment.

What will happen if I do not want to carry on with the study?

You are free to withdraw from the study at any time and this will not affect your care. You can either withdraw completely or choose to keep in contact with us to let us know your progress. Information collected earlier in the study may still be used.

What if there is a problem?

If you have concerns about any aspect of this study, you should ask to speak to the research coordinator who will do her best to answer your questions (contact numbers below).

If you remain unhappy and wish to complain formally, you can do this through the NHS Patient Advisory and Liaison Service (PALS) (Tel: 0800 389 8391; Email: pals@sbpct.nhs.uk). In the unlikely event that something does go wrong and you are harmed during the research and this is due to someone's negligence then you may have grounds for compensation against the University of Birmingham but you may have to pay your legal costs. The normal National Health Service complaints mechanisms will still be available to you.

Will my taking part in the study be confidential?

Yes. We will follow ethical and legal practice and all information about you will be handled in confidence. The study information will only be seen by the research team and will be stored in accordance with the Data Protection Act at the University of Birmingham. The study data may also be looked at by representatives of regulatory authorities and by authorized people to check that the study is being carried out correctly. All those associated with the study will have a duty of confidentiality to you as a research participant.

Will my GP be told that I am in a study?

Yes, we will inform your GP of your involvement in the study.

What will happen to any samples I give?

During study visits, extra blood and urine samples will be taken from you. These will be labelled with your study number. These samples will be anonymised and kept in a locked secure room within the University of Birmingham. Access will be restricted to the study researchers only. At the end of the study, these extra samples will be kept for 5 years and might be used for other future ethically approved studies.

Will any genetic tests be done?

No genetic tests will be performed.

What will happen to the results of the research study?

The results of the research will be published in international, peer-reviewed scientific journals. The results will also be available on the website www.clinicaltrials.gov. You will not be identifiable in any report or publication.

Who is organising and funding the research?

The study is organised by the Primary Care Clinical Research and Trials Unit (PC-CRTU) at the University of Birmingham and is funded by the National Institute for Health Research.

Who has reviewed the study?

Before deciding whether to fund the study, the National Institute for Health Research asked the opinion of independent expert. This study has also been reviewed and approved by West Midlands Research Ethics Committee.

What if I have more questions or do not understand something?

We will be pleased to answer any questions you may have or clarify things you do not understand. You can contact the STOP-CKD team on Tel: **0800 9230329**

If you wish to discuss the study with a doctor who is not directly involved with the study you may contact Dr Lukas Foggensteiner on Tel: 0121 371 5841.

What happens now if I decide to take part?

If you decide that you would like to participate in this study, we ask you to complete the attached form with your details and send it back to us in the envelope provided. You may also contact us by telephone or e-mail to inform us of your decision to participate. The STOP-CKD research team will then contact you to arrange a convenient day to attend your GP surgery.

Further information and contact details:

If you have any concerns or questions about the study, please contact:

STOP-CKD Research Team
Primary Care Clinical Research and Trial Unit,
University of Birmingham
Edgbaston
Birmingham, B15 2TT
Tel: 0800 923 0329
Email: stopckd@contacts.bham.ac.uk

Thank you very much for taking the time to consider taking part in our research.

If you decide that you would like to participate in this study, could you please kindly complete the attached form with your details and send it back to us in the envelope provided.

Please return to:
STOP-CKD Research Team,
Primary Care Clinical Research and Trial Unit,
University of Birmingham
Edgbaston
Birmingham
B15 2TT

Dear Dr Ferro

Name: ……………………………………………………………… Tel: ………………………………………………

Address: ……………………………………………………………… Mobile: ………………………………….

………………………………………………………………

……………………………………………………………… Postcode: ………………………………..

Signature: ……………………………………………………………… Date: __ __/__ __/__ __ __ __

Please tick if applicable:

□ **I am interested in participating in this study. I would be happy for a member of the study team to contact me to arrange a date to attend.**

□ **I do NOT wish to be involved in this study.**

*If you have decided **not** to take part in this study, it would be very useful if you could tell us your reasons to help us to improve other research studies in the future. We would be grateful if you could complete the following **voluntary** questionnaire and return it in the enclosed prepaid envelope:*

I am: Male ☐ Female ☐ My date of birth is: __ __/__ __/__ __ __ __

I do not wish to take part in this study for the following reason(s): *Tick as many as applicable*

- □ I do not have time to take part in the study
- □ I do not wish to take a new medication
- □ I do not wish to have further blood tests
- □ I do not wish to be part of a research trial
- □ Kidney problems are of no concern to me
- □ I am unable to attend the surgery
- □ I do not want to give a reason
- □ Other (Please specify): ………………………………

Please tick if applicable:

□ **I am interested to take part in an INTERVIEW study to discuss my reasons further**

We are carrying out a separate interview study to find out people's view of research study in kidney disease in the community. If you are interested, we will send out further information on the INTERVIEW study to you.

□ **I am NOT interested to take part in an INTERVIEW study to discuss my reasons further**

If you wish to give further information, please do so below:

………

………
