# Supplementary material for: Spironolactone to prevent cardiovascular events in early-stage chronic kidney disease (STOP-CKD): study protocol for a randomized controlled pilot trial
Source: Trials. 2014 May 6;15:158. doi: 10.1186/1745-6215-15-158 (PMC4113230; doi:10.1186/1745-6215-15-158)
Supplement: Additional file 2 — Consent form, part 1, version 2.2. [file 1745-6215-15-158-S2.doc]

| **Site ID:** |  |  |  |  |  |  |
| --- | --- | --- | --- | --- | --- | --- |
| **Patient ID:** |  |  |  |  |  |  |
| **Patient Initials:** |  |  |  |  |  |  |

|  |
| --- |

| 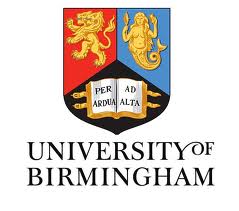 | STOP-CKD | 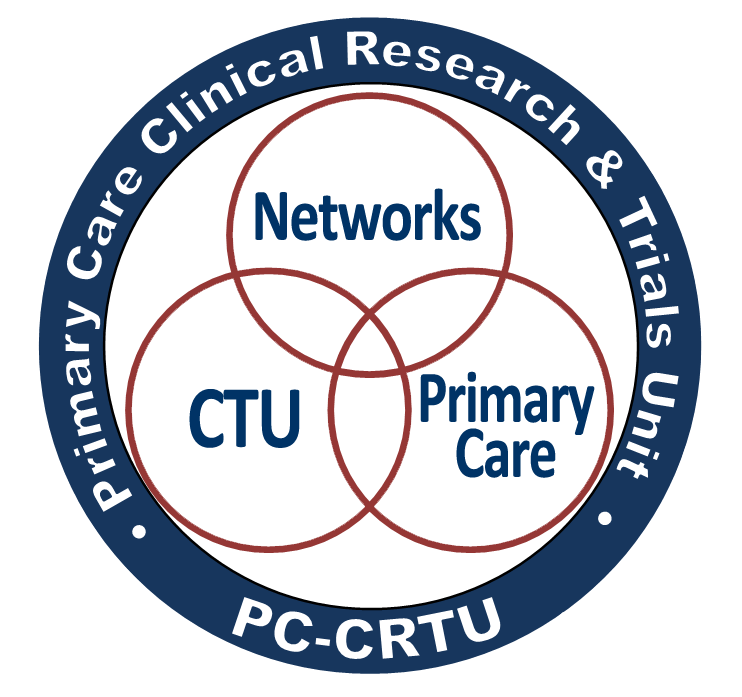 |
| --- | --- | --- |

**Spironolactone to Prevent Cardiovascular Events in**

**Early Stage Chronic Kidney Disease: A Pilot Trial**

**Patient Consent Form (Part 1)** Version 2.2 20/06/2013

**Please initial each box if you agree with the statement:**

1. I confirm that I have read and understood the information sheet dated 20/06/2013 (version 2.2) for the above study. I have had the opportunity to consider the information, to ask questions and have had these answered satisfactorily.
2. I give permission for my name to be given to the trials office when I am registered on the STOP-CKD study and for movement of my personal data from my GP Practice to the University of Birmingham.
3. I understand that relevant sections of my medical notes and data collected during the study may be looked at by individuals from the sponsor, regulatory authorities or from University of Birmingham where it is relevant to my taking part in this research. I give permission for these individuals to have access to my records. However, I understand that I will not be identified by name in any reports or publications resulting from this study.
4. I agree to donate an initial blood and urine sample for lab testing to determine eligibility for the study and understand that I may not be suitable to take part as detailed in the information sheet. I agree that samples taken will be stored for the duration of the study and 5 years after the end of the study which may be used for other future ethically approved studies.
5. I agree to my GP being informed of my participation in the study
6. I understand that my participation is voluntary and that I am free to withdraw at any time without giving a reason without my medical or legal rights being affected. I agree to take part in the above study

**………………………………… …………………………….. ……………………….**

Name of patient Date Signature

**………………………………… …………………………….. ………………………….**

Name of person taking consent Date Signature

**………………………………… …………………………….. ……………………….**

Name of Researcher Date Signature
