# Supplementary material for: Spironolactone to prevent cardiovascular events in early-stage chronic kidney disease (STOP-CKD): study protocol for a randomized controlled pilot trial
Source: Trials. 2014 May 6;15:158. doi: 10.1186/1745-6215-15-158 (PMC4113230; doi:10.1186/1745-6215-15-158)
Supplement: Additional file 3 — Consent form, part 2, version 2.2. [file 1745-6215-15-158-S3.doc]

| **Site ID:** |  |  |  |  |  |  |
| --- | --- | --- | --- | --- | --- | --- |
| **Patient ID:** |  |  |  |  |  |  |
| **Patient Initials:** |  |  |  |  |  |  |

| 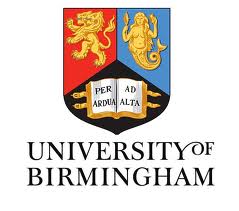 | STOP-CKD | 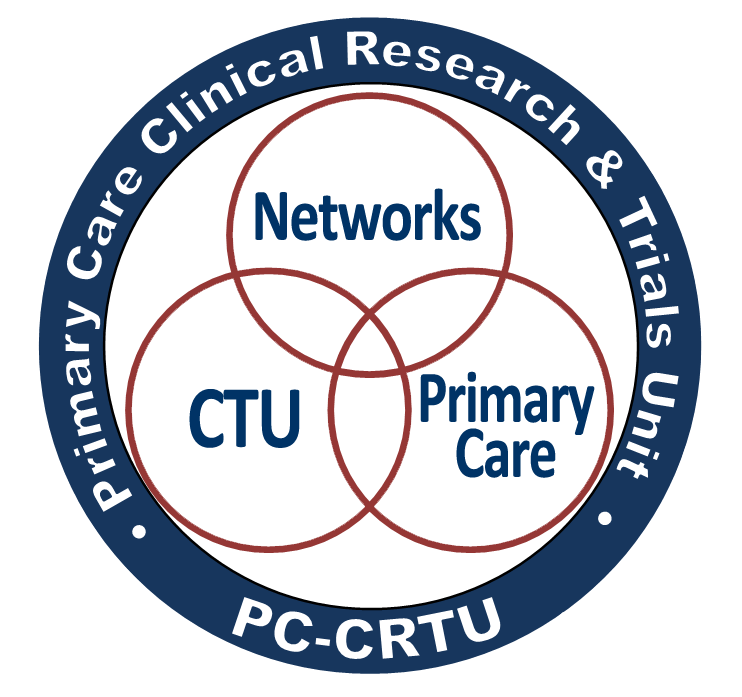 |
| --- | --- | --- |

**S**pironolactone **to P**revent **C**ardiovascular Events in

Early Stage **C**hronic **K**idney **D**isease: A Pilot Trial

**Patient Consent Form (Part 2)** Version 2.2 20/06/2013

**Please initial each box if you agree with the statement:**

1. I confirm that I have read and understood the information sheet dated 20/06/2013 (version 2.2) for the above study. I have had the opportunity to consider the information, to ask questions and have had these answered satisfactorily.
2. I give permission for my name to be given to the trials office when I am registered on the STOP-CKD study and for movement of information collected about me for the trial from my GP Practice to the University of Birmingham.
3. I understand that relevant sections of my medical notes and data collected during the study may be looked at by individuals from the sponsor, regulatory authorities or from University of Birmingham where it is relevant to my taking part in this research. I give permission for these individuals to have access to my records. However, I understand that I will not be identified by name in any reports or publications resulting from this study.
4. I understand participation in this study is a 46 weeks commitment. I agree to attend clinics and donate blood and urine samples for study as detailed in the information sheet. I agree that samples taken will be stored for the duration of the study and 5 years after the end of the study which may be used for other future ethically approved studies.
5. I understand that only specific pharmacies have been recruited to participate in the study and I agree to be directed to these pharmacies to obtain the trial medication prescribed.
6. I agree to my GP being informed of my participation in the study.
7. I understand that my participation is voluntary and that I am free to withdraw at any time without giving a reason without my medical or legal rights being affected. I agree to take part in the above study.

**………………………………… …………………………….. ……………………….**

Name of patient Date Signature

**………………………………… …………………………….. ……………………….**

Name of person taking consent Date Signature

**………………………………… …………………………….. ……………………….**

Name of Researcher Date Signature
