# Supplementary material for: Spironolactone to prevent cardiovascular events in early-stage chronic kidney disease (STOP-CKD): study protocol for a randomized controlled pilot trial
Source: Trials. 2014 May 6;15:158. doi: 10.1186/1745-6215-15-158 (PMC4113230; doi:10.1186/1745-6215-15-158)
Supplement: Additional file 4 — Medication monitoring questionnaire. [file 1745-6215-15-158-S4.docx]

Medication monitoring questionnaire

| **Side Effect** | **Yes** | **No** |
| --- | --- | --- |
| Nausea |  |  |
| Vomiting |  |  |
| Abdominal Discomfort |  |  |
| Diarrhoea |  |  |
| Black Discoloured Stool |  |  |
| Tiredness |  |  |
| Headache |  |  |
| Confusion |  |  |
| Drowsiness |  |  |
| Dizziness/ Imbalance |  |  |
| Breast swellings |  |  |
| Breast pain |  |  |
| Menstrual (period) disturbance |  |  |
| Change in libido |  |  |
| Excessive hair growth |  |  |
| Unwanted hair growth |  |  |
| Hair loss |  |  |
| Leg cramps |  |  |
| Rash |  |  |
| Joint pain |  |  |
| Others: (Please comment) |  | |
