# Supplementary material for: Spironolactone to prevent cardiovascular events in early-stage chronic kidney disease (STOP-CKD): study protocol for a randomized controlled pilot trial
Source: Trials. 2014 May 6;15:158. doi: 10.1186/1745-6215-15-158 (PMC4113230; doi:10.1186/1745-6215-15-158)
Supplement: Additional file 5 — Data monitoring committee charter. [file 1745-6215-15-158-S5.docx]

Data Monitoring and Ethics Committee Charter

Spironolactone to Prevent Cardiovascular Events in Early Stage Chronic Kidney Disease: A Pilot Trial

Ethics Ref: 12/WM/0168

EudraCT Number: 2012-002672-13

Sponsor: University of Birmingham

Funder: NIHR Research for Patient Benefit Grant PB-PG-0110-21226

Chief Investigator: Dr Charles Ferro, Department of Infection and Immunity

Trial Co-ordinator: Mrs Val Redman, Department of Primary Care

Statistician: Prof Nick Freemantle, Professor of Clinical Epidemiology and Biostatistics, University College London

Date: November 17th, 2013

# Introduction

This study is a prospective randomised double-blind placebo-controlled pilot study examining the actions and safety of spironolactone on arterial stiffness in patients with chronic kidney disease stage 3 in the community setting.

# Scope of the charter

The purpose of this document is to describe the roles and responsibilities of the independent DMC for the STOP CKD trial, including the timing of meetings, methods of providing information to and from the DMC, frequency and format of meetings, statistical issues and relationships with other committees.

# Roles and responsibilities

## Aims of the committee

To safeguard the interests of trial participants, monitor the safety and efficacy of the interventions during the trial, and monitor the overall conduct of the clinical trial.

## Terms of reference

The DMC should receive and review the progress and accruing data of this trial and provide advice on the conduct of the trial to the Trial Steering Committee (TSC).

The DMC should inform the Chair of the TSC if, in their view the results are likely to convince a broad range of clinicians, including those supporting the trial and the general clinical community, that on balance one trial arm is clearly indicated or contraindicated for all participants or a particular category of participants, and there was a reasonable expectation that this new evidence would materially influence patient management.

## Specific roles of the DMEC

The DMC will have an independent chair with authority to advise early termination of the trial in the event of safety concerns or futility whether through poor recruitment, lack of events, or lack of any treatment effect. All committees will convene regularly prior to, during, and following the trial.

Together, the responsibilities of the committee are:

- To safeguard the safety, rights and well-being of the trial participants.
- To systematically monitor the trial data and review any analysis as outlined in the Statistical Analysis Plan or as requested by the TSC.
- To make recommendations to the TSC as to whether the trial is operating as expected or if there are any ethical or safety reasons why the trial should not continue.
- To consider data emerging from other related studies and its potential impact on the trial, if requested by the TSC.
- To pick up any trends, such as increases in un/expected events, and take appropriate action.
- To seek additional advice or information from investigators where required.
- To act or advise, through the Chairman or other consultant, on incidents occurring between meetings that require rapid assessment.

## Before or early in the trial

The DMC will meet in the first year of trial recruitment. The specific purpose of the initial meeting will be to;

- discuss the trial
- discuss the DMEC analysis plan
- allow opportunity for the DMC to clarify any aspects of the trial with the principal investigators
- agree to the DMC Charter

## Composition

The members of the DMEC for this trial are;

| Name | DMEC Role | Title | Affiliation |
| --- | --- | --- | --- |
| Prof Ian Squire | Chair | Professor of Cardiology | University of Leicester |
| Dr Kelvin Jordan | Ordinary Member | Reader in Biostatistics | Keele University |
| Prof Kamlesh Kunti | Ordinary member | Professor of Primary Care | University of Leicester |

The trial statistician (Nick Freemantle) in collaboration with the Trial Co-ordinator (Mrs Val Redman) will produce the report to the DMC and will participate in DMC meetings. The report will be circulated two weeks prior to the scheduled DMC meeting.

The Chief Investigator (Dr Charles Ferro), the Trial Co-ordinator (Mrs Val Redman) and Chair of the TSC will remain blind to outcome data summarised according to randomised allocation and will only be invited to attend the open sessions of the DMEC.

# Relationships

The Chair of the DMC will report back to the Chair of the TSC with any recommendations regarding the conduct of the trial. All decisions regarding the conduct of the trial will be made by the TSC.

Members of the DMC will be reimbursed for accommodation and travel, but will not receive any payment for sitting on the trial DMC.

Members of the DMC will disclose any competing interests and will complete a competing interests form lodged with the Primary Care Clinical Trials Unit (PC-CRTU).

# Organisation of DMEC meetings

The DMC will meet annually, or as required.

Ideally, meetings will be face-to-face, however, due to the geographical distribution of the DMC members, it is likely that some meetings will be held by teleconference.

## Proposed format:

1. Open session: Introduction and any “open” parts of the report
2. Closed session: DMC discussion of “closed” parts of the report

and, if necessary,

1. Open session: Discussion with other attendees on any matters arising from the previous session(s).
2. Closed session: extra closed session

# Trial documentation and procedures to ensure confidentiality and proper communication

## Content of material to be available in open sessions

Accumulating information relating to recruitment and data quality will be presented. This will include number of patients eligible, number of patients randomised, completeness of adjudicated outcome data, and number of ineligible patients randomised. This information will be reported overall and by centre.

The DMC may wish to include outcome data pooled across randomised groups but not presented by individual treatment groups.

## Content of material to be available in closed sessions

Efficacy and safety data will be presented by randomised group.

## Blinding of DMEC

It is proposed that the DMC are not blinded and will be able to review outcome data according to randomised group.

The closed session DMC report is confidential and will only be seen by the DMC and the trial statistician. The DMC and the trial statistician will not share this information with anyone outside of the DMC, including the Chief Investigator.

DMC members should store the papers safely after each meeting so they may check the next report against them. Following publication of the trial, DMC members should destroy all DMC reports.

# Decision making

## Guidelines for stopping

The DMC will have authority to advise early termination of the trial in the event of safety concerns or futility (whether through poor recruitment, lack of events, or lack of any treatment effect).

## Making recommendations

Effort should be made for all members to attend. The PC-CRTU will try to ensure that a date is chosen to achieve this. Members who cannot attend in person should be encouraged to attend by teleconference. The DMEC will be deemed quorate if 3 members are present (to include at least the statistician and one clinician).

Members of the DMEC who are unable to be present at the meeting may pass comments to the DMEC Chair for consideration during the discussions.

If the DMEC is considering recommending major action after such a meeting the DMEC Chair should talk with the absent members as soon after the meeting as possible to check they agree. If they do not, a further teleconference should be arranged with the full DMEC.

If a member does not attend a meeting, it should be ensured that the member is available for the next meeting. If a member does not attend a second meeting, they should be asked if they wish to remain part of the DMEC. If a member does not attend a third meeting, they should be replaced.

# Reporting

A letter detailing the recommendations of the DMEC should be sent to the TSC and copied to the PC-CRTU within 3 weeks of the meeting.

Minutes of the DMC meetings will be drafted by the trial statistician and circulated once they have been signed off by the Chair.

If the DMC has serious problems or concerns with the TSC decision a meeting of these groups should be held. The information to be shown would depend upon the action proposed and the DMC’s concerns. Depending on the reason for the disagreement confidential data will often have to be revealed to all those attending such a meeting. The meeting should be chaired by a senior member of the trials office staff or an external expert who is not directly involved with the trial.

# After the trial

After the trial it is suggested that a meeting with DMC members and the Chief Investigator convened to give advice about data interpretation and have opportunity to read and comment on publications before submission.

DMC members should be named and their affiliations listed in the main report, unless they explicitly request otherwise. A brief summary of the timings and conclusions of DMC meetings should be included in the body of the report.

The DMC may discuss issues from their involvement in the trial after the primary trial results have been published.

# Publication of results

The DMC will expect the results will be reported in a correct and timely manner.

# Declaration of competing interests

## Potential competing interests DMC members

The avoidance of any perception that members of a DMC may be biased in some fashion is important for the credibility of the decisions made by the DMC and the integrity of the trial.

Possible competing interests should be disclosed. In many cases simple disclosure up front should be sufficient. Otherwise, the DMC member should remove the conflict or stop participating in the DMC.

Potential competing interests

- Stock ownership or transaction in any commercial companies involved
- Consulting arrangements with the sponsor
- Frequent speaking engagements on behalf of the intervention
- Career tied up in a product or technique assessed by trial
- Personal participation or emotional involvement in the running of the trial
- Intellectual conflict, e.g. strong prior belief in the trial’s experimental arm
- Involvement in regulatory issues relevant to trial procedures
- Investment (financial or intellectual) in competing products
- Involvement in the publication
- Other:_______________________________________________________

Please complete and return to Mrs Val Redman at the PC-CRTU.

| No, I have no competing interests to declare |  |
| --- | --- |
| Yes, I have competing interests to declare |  |

Please provide details of any competing interests;

_____________________________________________________________________

_____________________________________________________________________

Name: …………………………………………………….

Signed: …………………………………………………… Date: …………………….
